# A biochemical comparison of the lung, colonic, brain, renal, and ovarian cancer cell lines using ^1^H-NMR spectroscopy

**DOI:** 10.1042/BSR20194027

**Published:** 2020-04-23

**Authors:** Cong Hu, Zhigang Liu, Hailin Zhao, Lingzhi Wu, Qingquan Lian, Daqing Ma, Jia V. Li

**Affiliations:** 1 Department of Anesthesiology, The Second Affiliated Hospital and Yuying Children’s Hospital of Wenzhou Medical University, Wenzhou, Zhejiang, China; 2 Division of Anesthetics, Pain Medicine and Intensive Care, Department of Surgery and Cancer, Faculty of Medicine, Chelsea and Westminster Hospital, Imperial College London, United Kingdom; 3 Department of Metabolism, Digestion and Reproduction, Faculty of Medicine, Imperial College London, United Kingdom

**Keywords:** colonic cancer, glioma, lung cancer, metabolomics, NMR spectroscopy, renal cancer

## Abstract

Cancer cell lines are often used for cancer research. However, continuous genetic instability-induced heterogeneity of cell lines can hinder the reproducibility of cancer research. Molecular profiling approaches including transcriptomics, chromatin modification profiling, and proteomics are used to evaluate the phenotypic characteristics of cell lines. However, these do not reflect the metabolic function at the molecular level. Metabolic phenotyping is a powerful tool to profile the biochemical composition of cell lines. In the present study, ^1^H-NMR spectroscopy-based metabolic phenotyping was used to detect metabolic differences among five cancer cell lines, namely, lung (A549), colonic (Caco2), brain (H4), renal (RCC), and ovarian (SKOV3) cancer cells. The concentrations of choline, creatine, lactate, alanine, fumarate and succinate varied remarkably among different cell types. The significantly higher intracellular concentrations of glutathione, myo-inositol, and phosphocholine were found in the SKOV3 cell line relative to other cell lines. The concentration of glutamate was higher in both SKOV3 and RCC cells compared with other cell lines. For cell culture media analysis, isopropanol was found to be the highest in RCC media, followed by A549 and SKOV3 media, while acetone was the highest in A549, followed by RCC and SKOV3. These results demonstrated that ^1^H-NMR-based metabolic phenotyping approach allows us to characterize specific metabolic signatures of cancer cell lines and provides phenotypical information of cellular metabolism.

## Introduction

Cancer is the second leading cause of global mortality with approximately 9.6 million deaths in 2018. Some types of cancers including lung and colorectal cancer are among the most common causes of the mortality, accounting for 18% and 8.9% of the total cancer death, respectively. It was reported in 2018 that lung, colorectal, kidney, brain, and ovarian cancer contributed to 12.3%, 10.6%, 2.4%, 1.7%, and 1.7% of the total number of cancer cases, respectively [25]. Although brain cancers only accounted for 1.7% of the total cancer cases in 2018, it is the second most common cancers in children, contributing 26% of childhood cancers [1,2].

To explore cellular or molecular mechanisms, responses to therapies and drug discovery and development of cancers, cancer cell lines have been widely used and served as the workhorse for cancer research. However, it has been recently reported that single cell-derived clones showed continuous instabilities, leading to the heterogeneity of the cell lines, altering the drug responses, and dysregulation of xenobiotic metabolism [3]. This suggested that cell line-based research should be documented with the extent, origins and consequences of genetic variation of the cell lines to improve the reproducibility of the cancer research. Furthermore, other molecular profiling approaches including transcriptomics, chromatin modification profiling, and proteomics have been used to evaluate the phenotypic characteristics of cell lines. However, these do not reflect the functional consequences at the metabolic level.

Metabolic phenotyping is a powerful tool to profile the biochemical composition of the cell lines and explore the metabolic pathways affected by mutations, transcriptional regulators, and xenobiotics. Both nuclear magnetic resonance (NMR) spectroscopy and mass spectrometry are widely used to study metabolic phenotyping. Although mass spectrometry has high sensitivity, NMR spectroscopy is more reproducible and is non-destructive to samples [4]. Hence, NMR spectroscopy can be a robust tool to evaluate the metabolic status, complementing genomic, transcriptomic, and proteomic profiling of cell lines. ^1^H-NMR spectroscopy-based approach has previously been applied to study the metabolic responses of cancer cell lines, such as lung (A549) and colon (Caco2), to xenobiotics and mycotoxins [5,6]. However, the metabolite composition of these commonly used cell lines has not yet been compared directly to provide a broad insight into the metabolic differences among the cells.

In the present study, ^1^H-NMR spectroscopy was used to profile five cancer cell lines, namely, lung (A549), colonic (Caco2), brain (H4), renal (RCC), and ovarian (SKOV3) cancer cells to detect broad metabolic fingerprints. These fingerprints can be used to compare with other molecular profiles, which may further our understanding of the mechanisms and pathways responsible for the development and progression of different kinds of cancer.

## Materials and methods

### Cell culture and sample collection

Lung (A549), colonic (Caco2), brain (H4), renal (RCC), and ovarian (SKOV3) cancer cells were purchased from ECACC (Wiltshire, U.K.). To optimize the number of cells per type of cell lines for ^1^H-NMR spectroscopic analysis, cells were seeded in 35, 60, or 100 mm Petri dishes and cultured at 37°C with 5% CO_2_ for 2 (A549, H4 and RCC) or 3 (Caco2, SKOV3) days to reach approximately 1, 5, or 10 million cells. The cell number was counted with flow cytometry after the counting beads were added in the cell suspension. A549, RCC, and SKOV3 cell lines were cultured in RPMI media (ThermoFisher, Paisley, U.K.), while Caco2 and H4 cell lines were cultured in DMEM media (ThermoFisher, Paisley, U.K). The cultured samples were centrifuged at 1500 ***g*** at 4°C for 5 min. The supernatant (1 ml) was transferred into 1.5 ml Eppendorf tubes and stored at −80°C, while the cell pellets were washed with PBS three times before storing at −80°C.

### Metabolite extraction of the cell pellets

Cell pellets were placed into a bead beater tube (STARLAB Science Laboratory, Hamburg, Germany) containing 0.1 g sterile beads with a diameter of 0.1 mm and 1.5 ml of the pre-chilled mixture of methanol (Thermo Fisher Scientific, Paisley, U.K.) and water (MeOH:H_2_O, v:v, 1:1). The tubes were placed in a bead beater (Bertin Instruments, Montigny-le-Bretonneux, France) to homogenize the samples using two cycles of 6500 Hz for 40 s with 5 min on dry ice between cycles. The samples were then centrifuged at 10,000 ***g*** at 4°C for 10 min, and the supernatants were transferred to new Eppendorf tubes before drying at 45°C overnight and stored at −40°C.

### Sample preparation for ^1^H-NMR spectroscopy

The dry cell extract samples were resuspended in 210 μl of potassium phosphate buffer (pH = 7.4) containing deuterium oxide (D_2_O) for magnetic field lock, 0.005% 3-(trimethylsilyl)-[2,2,3,3-^2^H_4_]-propionic acid sodium salt (TSP) for the spectral calibration, 0.075 M KH_2_PO_4_, and 0.1 mM sodium azide (NaN_3_). The resulting mixture was centrifuged at 20,817 ***g*** for 10 min, and 180 μl supernatant was transferred to an NMR tube (Bruker Corporation, Rheinstetten, Germany) with an outer diameter of 3 mm pending ^1^H-NMR spectral acquisition.

The cell media were defrosted and centrifuged at 18,000 ***g*** for 10 min. A total of 540 μl supernatant was mixed with 60 μl potassium phosphate buffer containing D_2_O, 0.1% TSP, 1.5 M KH_2_PO_4_, and 2 mM NaN_3_. The mixture was transferred to an NMR tube with an outer diameter of 5 mm pending ^1^H-NMR spectral acquisition.

### ^1^H-NMR spectroscopy

^1^H-NMR spectra of cell extracts and media samples were obtained using a Bruker 600 MHz spectrometer (Bruker Corporation, Rheinstetten, Germany) at the operating ^1^H frequency of 600.13 MHz at a temperature of 300 K. A standard NMR pulse sequence (recycle delay-90°-*t*_1_-90°-*t*_m_-90° acquisition) was applied to acquire ^1^H-NMR spectral data (*t*_1_ = 3 μs, *t*_m_ = 100 ms). The water peak suppression was achieved using selective irradiation during a recycle delay of 4 s and *t*_m_. A 90° pulse was adjusted to ∼10 μs. A total of 64 scans for cell extracts and 32 scans for cell media were collected into 64 k data points with a spectral width of 20 ppm. Two-dimensional (2-D) ^1^H-^1^H correlation spectroscopy (COSY), ^1^H-^1^H total correlation spectroscopy (TOCSY), and heteronuclear single quantum coherence spectroscopy (HSQC) were acquired on the selected cell and media samples for to aid in metabolite identification.

### Multivariate statistical analysis of the spectral data

^1^H-NMR spectra obtained from cell extracts and media samples were phased, referenced to TSP at δ^1^H 0.00 and baseline-corrected in TopSpin 4.0.3 (Bruker Corporation, Rheinstetten, Germany). MATLAB software R2018a (MathWorks, Cambridge, U.K.) programming language was used to import and process the NMR spectral data. Water peak regions of the cell extract (δ^1^H 4.74–4.85) and cell media (δ^1^H 4.7–5) spectra were deleted to minimize the effect of the disordered baseline. Regions containing only noise in the cell extract (δ^1^H 0–0.5, 9.5–10) and cell media (δ^1^H 0–0.3) spectra were removed. Two cellular extract samples from 5 million H4 cell group and one media sample from 5 million RCC cell group were excluded due to extremely low intensities of signals. The remaining spectra data from 1, 5, and 10 million cells were normalized using a probabilistic quotient normalization method separately [7]. Principal component analysis (PCA) and orthogonal projection to latent structures-discriminant analysis (OPLS-DA) were carried out based on the unit variance-scaled datasets in SIMCA-15 (Umetrics, Sartorius Stedim Biotech) and MATLAB (The MathWorks, Inc.) software. The PCA, an unsupervised method, can reduce data dimensions to several principal components, which allows the visualization of data variations; in other words, it can describe intrinsic similarities or differences of the data [8]. In contrast, OPLS-DA is a supervised method, which requires the sample class information (e.g. control vs. intervention) and shows the metabolic differences between the classes. In an OPLS-DA model, R^2^X and R^2^Y represent the variation explained by the model in X and Y matrices, respectively. Q^2^Y represents predictability of the model and a good model usually has a Q^2^Y > 0.5 [8]. A permutation test of the OPLS-DA model was also carried out to generate a *P* value. Models with *P* < 0.05 are considered as valid OPLS-DA models.

## Results

### Characterizing the biochemical composition of A549, Caco2, H4, RCC, and SKOV3 cell lines

The median ^1^H-NMR spectra of the cell extracts obtained from 1, 5, and 10 million cells per cell type are shown in Supplementary Figure S1. By visualizing the spectra, peak intensities increase proportionally as the number of cells increases from 1 to 10 million. The metabolite assignment from cell extracts and media samples are listed in the Supplementary Table S1. Five million cells produced a better quality of the spectra with 64 scans than one million cells. A total of 34 metabolites were identified from these cellular extracts and confirmed using 2-D ^1^H-^1^H COSY and ^1^H-^1^H TOCSY NMR spectra (Supplementary Figure S2); these metabolites included acetate, alanine, asparagine, aspartate, choline, creatine, formate, fumarate, glutamate, glutamine, glutathione, glycerol phosphocholine, glycine, histidine, hypoxanthine, isoleucine, lactate, leucine, lysine, methanol, methionine, myo-inositol, phenylalanine, phosphocholine, serine, succinate, taurine, threonine, trehalose, tryptophan, tyrosine, uracil, uridine, and valine.

**Figure 1:**
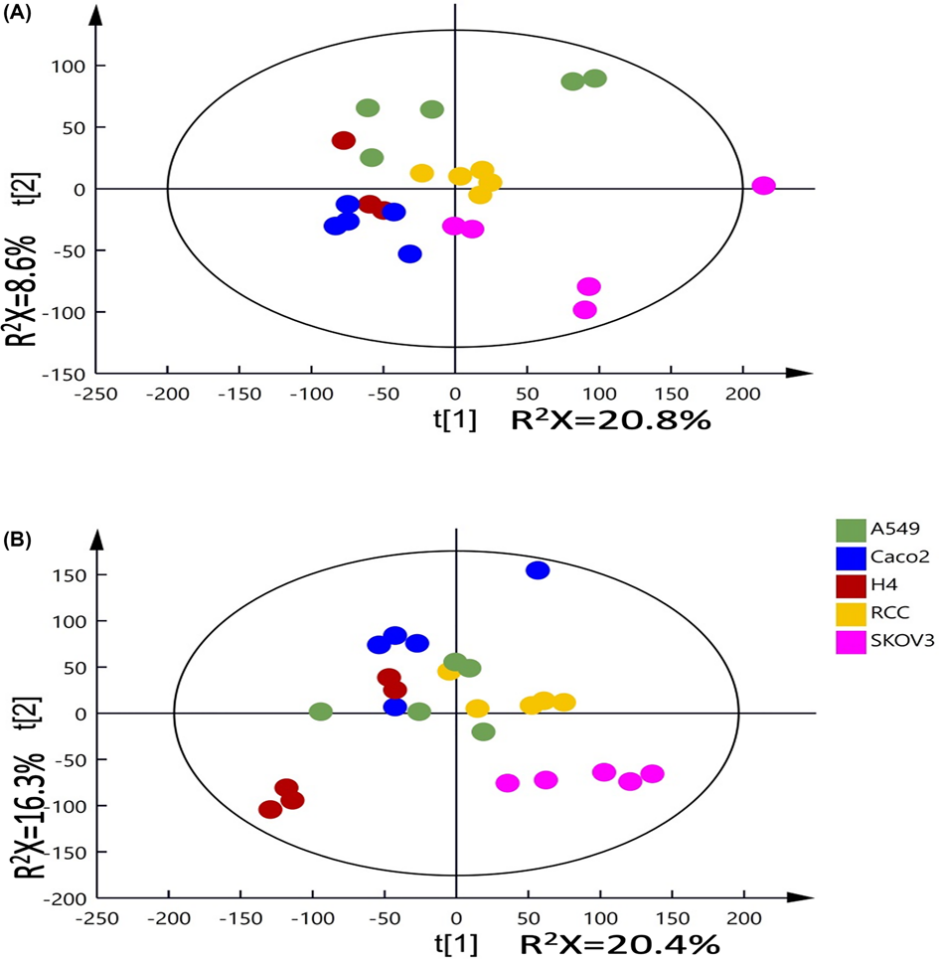
PCA scores plots of ^1^H-NMR spectra of cellular extracts from 5 and 10 million cells Principal components analysis (PCA) scores plots of ^1^H-NMR spectra of cellular extracts from 5 (**A**) and 10 (**B**) million cells. R^2^X represents the fraction of variation in the NMR spectral data modelled by each of the principal components (*t*[1] vs. *t*[2]). Five replicates per cell type except for H4 (5 million cells) with three replicates included.

The metabolic profiles obtained from 5 and 10 million cells were analyzed using unsupervised PCA analysis with three principal components (PC). The PCA scores plots of PC1 versus PC2 derived from both 5 and 10 million cells (Figure 1) show a grouping pattern based on the cell types, except for H4. This grouping pattern is clearer in the scores plot (PC1 versus PC2) with 10 million cells, while for 5 million cells it is clearer in the scores plot (PC2 versus PC3) (Supplementary Figure S3B).

**Figure 2:**
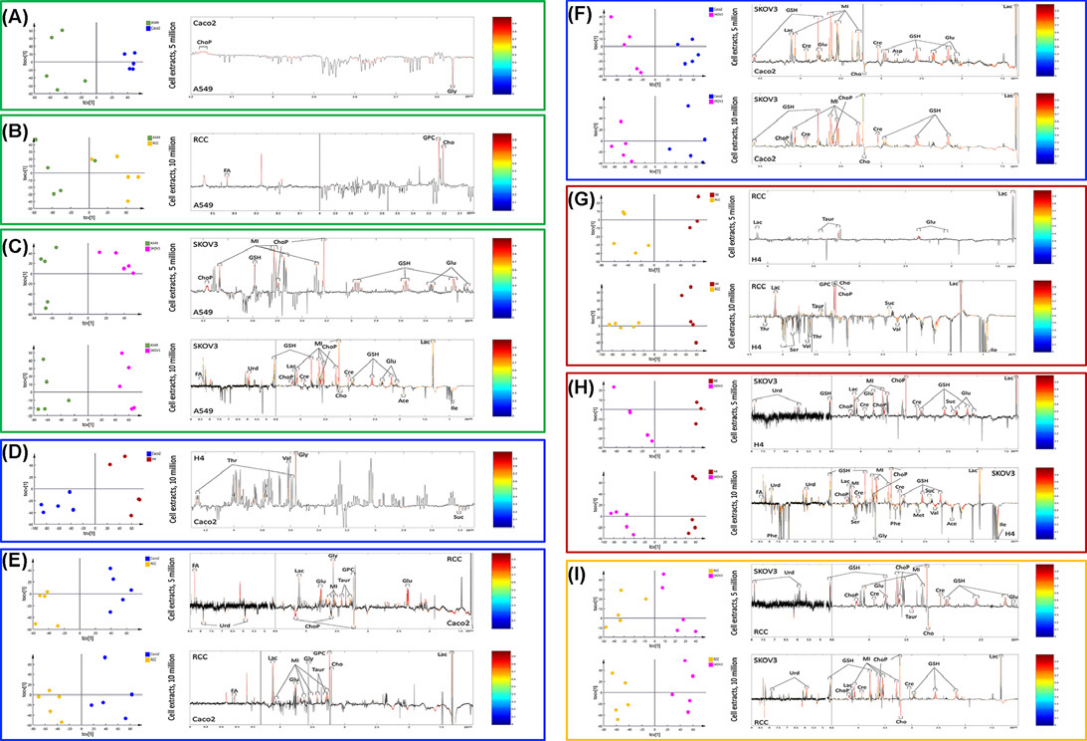
OPLS-DA cross-validated scores plots and loadings plots from ^1^H-NMR spectral data of the cell extract samples Orthogonal projection to latent structures-discriminant analysis (OPLS-DA) cross-validated scores plots (left panel) and the corresponding loadings plots (right panel) from ^1^H-NMR spectral data of the cell extract samples for comparisons as A549 versus Caco2 (**A**), RCC (**B**), or SKOV3 (**C**); Caco2 versus H4 (**D**), RCC (**E**), or SKOV3 (**F**); H4 versus RCC (**G**) or SKOV3 (**H**); RCC versus SKOV3 (**I**). Color bars in the loadings plot indicates the square of correlation coefficient values (*r^2^*). Black peaks indicate non-significant metabolites, whereas colourful peaks represent a statistical significance after Benjamini–Hochberg corrections (FDR *q*<0.05).

**Figure 3:**
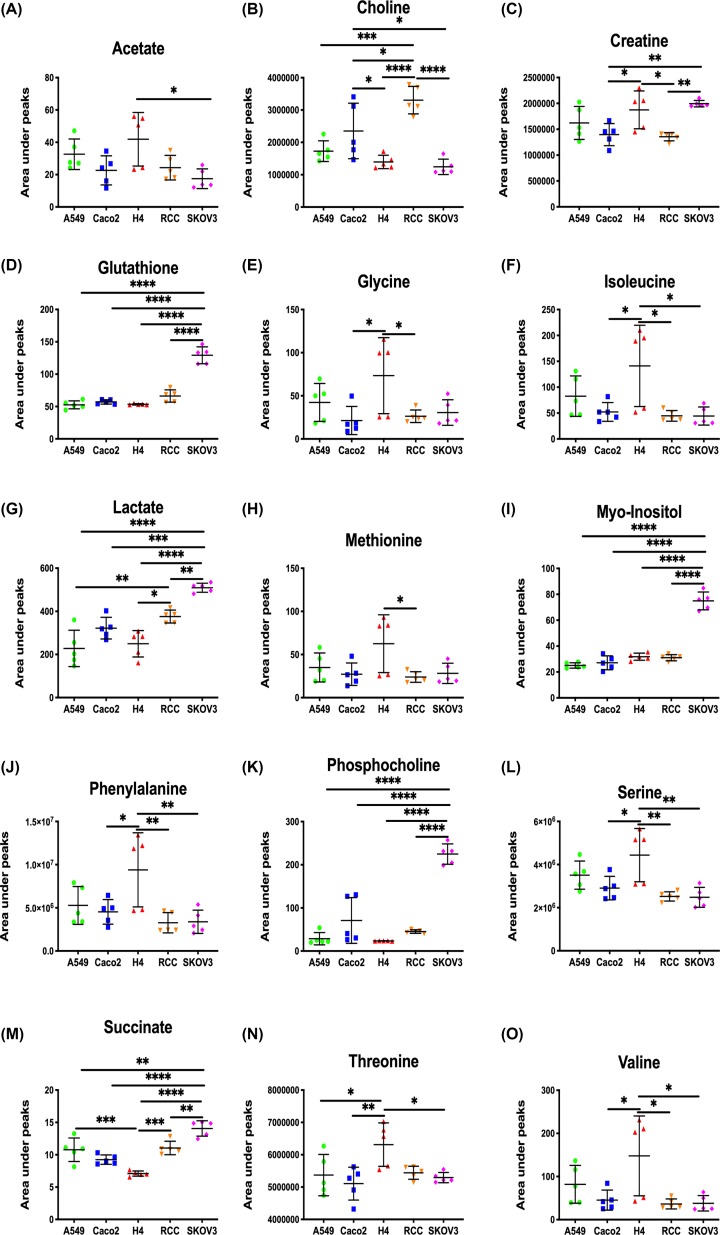
Peak integrals of the selected peaks from 15 metabolites from cell extracts of 10 million cells Peak integrals of the selected peaks from 15 metabolites from 10 million cells of A549, Caco2, H4, RCC, and SKOV3 cell extracts. The integrated values indicate relative concentrations of metabolites. (**A**) Acetate; (**B**) Choline; (**C**) Creatine; (**D**) Glutathione; (**E**) Glycine; (**F**) Isoleucine; (**G**) Lactate; (**H**) Methionine; (**I**) Myo-Inositol; (**J**) Phenylalanine; (**K**) Phosphocholine; (**L**) Serine; (**M**) Succinate; (**N**) Threonine; (**O**) Valine. Data were shown as mean ± SD, with Tukey’s multiple comparisons test; *P<0.05, **P<0.01, ***P<0.001, ****P<0.0001 (*n*=5).

Pair-wise comparisons between different cell types were carried out using OPLS-DA analysis with one predictive component and one orthogonal component. The R^2^X, Q^2^X, Q^2^Y, and permutation *P* values of these OPLS-DA models are summarized in Table 1. The loading plots from the significant OPLS-DA models and the metabolite changes observed in the pair-wise comparisons are shown in Figure 2 and Table 2. Peak integrals of 15 metabolites from 10 million cells of A549, Caco2, H4, RCC, and SKOV3 cell extracts are presented in Figure 3.

**Table 1:** Summary of the parameters of the OPLS-DA models based on the cell extracts comparing different cell types

Models	Cell number (million)	O-PLS-DA statistical parameters			
		R^2^X	R^2^Y	Q^2^Y	Permutation *P* value
A549 versus Caco2	5	45.22%	97.77%	0.77	0.008
	10	41.86%	94.79%	0.54	ns (0.063)
A549 versus H4	5	47.34%	86.10%	0.45	ns (0.098)
	10	47.45%	94.01%	0.7	0.004
A549 versus RCC	5	25.71%	99.32%	0.59	0.028
	10	37.42%	99.24%	0.71	0.001
A549 versus SKOV3	5	43.24%	98.85%	0.71	0.017
	10	42.67%	99.44%	0.87	0.001
Caco2 versus H4	5	48.74%	91.65%	0.7	0.001
	10	49.03%	97.98%	0.85	0.001
Caco2 versus RCC	5	35.99%	99.77%	0.79	0.001
	10	36.01%	98.75%	0.69	0.026
Caco2 versus SKOV3	5	43.94%	99.65%	0.75	0.01
	10	42.10%	98.91%	0.87	0.009
H4 versus RCC	5	48.27%	93.56%	0.67	0.012
	10	49.77%	98.74%	0.87	0.006
H4 versus SKOV3	5	45.19%	92.44%	0.7	0.006
	10	48.82%	99.35%	0.92	0.001
RCC versus SKOV3	5	38.25%	98.30%	0.59	0.018
	10	41.89%	99.50%	0.88	0.001

H4 cell (*n*=3); other cell types (*n*=5). Permutation *P* values were derived from 1000 permutes.

**Table 2:** Summary of the metabolites that are present in different concentrations in different cell types

Metabolites	δ^1^Η	*n*	Models	*r*	*P*	*q*
Acetate	1.92 (s)	10	A549 versus SKOV3	−0.86	1.28E-03	1.89E-02
			H4 versus SKOV3	−0.80	5.10E-03	3.39E-02
Aspartate	2.69 (m); 2.82 (dd); 3.90 (m)	5	Caco2 versus SKOV3	0.91	2.07E-04	1.15E-02
Choline	3.21 (s); 3.53 (m); 4.07 (m)	5	Caco2 versus SKOV3	−0.81	4.22E-03	4.53E-02
			RCC versus SKOV3	−0.88	8.43E-04	4.54E-02
		10	A549 versus RCC	0.91	2.17E-04	3.11E-02
			A549 versus SKOV3	−0.80	5.09E-03	3.91E-02
			Caco2 versus RCC	0.92	1.91E-04	1.37E-02
			Caco2 versus SKOV3	−0.82	3.43E-03	2.15E-02
			H4 versus RCC	0.90	4.42E-04	1.28E-02
			RCC versus SKOV3	−0.92	1.63E-04	4.94E-03
Creatine	3.04 (s); 3.93 (s)	5	Caco2 versus SKOV3	0.92	1.72E-04	1.09E-02
			H4 versus SKOV3	0.92	1.69E-04	1.95E-02
			RCC versus SKOV3	0.90	4.44E-04	3.16E-02
		10	A549 versus SKOV3	0.93	7.35E-05	4.18E-03
			Caco2 versus SKOV3	0.98	1.43E-06	4.84E-04
			H4 versus SKOV3	0.92	1.38E-04	3.02E-03
			RCC versus SKOV3	0.97	4.51E-06	7.07E-04
Formate	8.46 (s)	5	Caco2 versus RCC	0.83	2.73E-03	4.82E-02
		10	A549 versus RCC	0.91	2.28E-04	3.14E-02
			A549 versus SKOV3	0.92	1.80E-04	6.41E-03
			Caco2 versus RCC	0.89	5.33E-04	2.32E-02
			H4 versus SKOV3	0.87	1.13E-03	1.25E-02
Glutamate	2.07 (m); 2.12 (m); 2.36(m); 3.77 (m)	5	A549 versus SKOV3	0.94	5.69E-05	9.09E-03
			Caco2 versus RCC	0.94	6.71E-05	1.04E-02
			Caco2 versus SKOV3	0.86	1.46E-03	2.50E-02
			H4 versus RCC	0.97	5.69E-06	1.14E-02
			H4 versus SKOV3	0.91	2.45E-04	2.31E-02
			RCC versus SKOV3	0.96	8.08E-06	3.75E-03
		10	A549 versus SKOV3	0.93	1.18E-04	5.14E-03
			Caco2 versus RCC	0.91	2.69E-04	1.62E-02
Glutathione	2.17 (m); 2.56 (m); 2.96 (m); 3.78 (m); 4.56 (t)	5	A549 versus SKOV3	0.94	6.19E-05	9.46E-03
			Caco2 versus SKOV3	0.98	6.18E-07	1.36E-03
			H4 versus SKOV3	0.98	1.07E-06	1.35E-03
			RCC versus SKOV3	0.95	3.47E-05	6.85E-03
		10	A549 versus SKOV3	0.99	2.81E-09	7.43E-05
			Caco2 versus SKOV3	0.97	1.72E-06	4.91E-04
			H4 versus SKOV3	0.99	9.21E-08	1.79E-04
			RCC versus SKOV3	0.95	2.14E-05	1.63E-03
Glycerol phosphocholine	3.23 (s); 3.60 (dd); 3.68 (t); 3.72 (dd); 3.89 (m); 4.32 (t)	5	Caco2 versus RCC	0.89	6.40E-04	2.35E-02
		10	A549 versus RCC	0.92	1.92E-04	2.97E-02
			Caco2 versus RCC	0.89	5.02E-04	2.27E-02
			H4 versus RCC	0.94	4.23E-05	5.22E-03
Glycine	3.56 (s)	5	A549 versus Caco2	−0.91	2.70E-04	2.83E-02
			Caco2 versus RCC	0.92	1.93E-04	1.39E-02
		10	Caco2 versus H4	0.89	6.62E-04	1.73E-02
			Caco2 versus RCC	0.87	1.22E-03	3.63E-02
			H4 versus SKOV3	−0.79	6.93E-03	4.12E-02
Isoleucine	0.94 (t); 1.01 (d); 1.27 (m); 1.48 (m); 3.67 (m)	10	A549 versus SKOV3	−0.88	6.73E-04	1.34E-02
			H4 versus RCC	−0.86	1.60E-03	2.34E-02
			H4 versus SKOV3	−0.94	5.59E-05	1.70E-03
Lactate	1.33 (d); 4.11 (q)	5	Caco2 versus RCC	0.83	2.83E-03	4.91E-02
			Caco2 versus SKOV3	0.86	1.37E-03	2.43E-02
			H4 versus RCC	0.91	2.22E-04	4.54E-02
			H4 versus SKOV3	0.92	1.87E-04	2.01E-02
		10	A549 versus SKOV3	0.90	4.20E-04	1.03E-02
			Caco2 versus RCC	0.89	6.11E-04	2.49E-02
			H4 versus RCC	0.87	1.21E-03	2.07E-02
			H4 versus SKOV3	0.89	6.57E-04	8.63E-03
			RCC versus SKOV3	0.90	4.37E-04	8.78E-03
Methionine	2.14 (s); 2.16 (m); 2.65 (t); 3.86 (m)	10	H4 versus SKOV3	−0.81	4.50E-03	3.12E-02
Myo-inositol	3.28 (t); 3.53 (dd); 3.62 (t); 4.06 (t)	5	A549 versus SKOV3	0.90	3.84E-04	2.96E-02
			Caco2 versus RCC	0.88	6.85E-04	2.42E-02
			Caco2 versus SKOV3	0.85	1.80E-03	2.79E-02
			H4 versus SKOV3	0.91	3.10E-04	2.67E-02
			RCC versus SKOV3	0.90	4.62E-04	3.22E-02
		10	A549 versus SKOV3	0.99	1.21E-07	4.55E-04
			Caco2 versus RCC	0.92	1.80E-04	1.35E-02
			Caco2 versus SKOV3	0.98	1.15E-06	4.79E-04
			H4 versus SKOV3	0.93	7.52E-05	2.05E-03
			RCC versus SKOV3	0.97	5.34E-06	7.71E-04
Phenylalanine	3.13 (dd); 3.28 (dd); 3.98 (dd); 7.33 (m); 7.38 (m); 7.43 (m)	10	H4 versus SKOV3	−0.93	7.79E-05	2.10E-03
Phosphocholine	3.22 (s); 3.60 (m); 4.17 (m)	5	A549 versus Caco2	0.93	9.32E-05	1.84E-02
			A549 versus SKOV3	0.96	1.67E-05	4.28E-03
			Caco2 versus RCC	−0.92	1.65E-04	1.32E-02
			H4 versus SKOV3	0.96	6.51E-06	3.27E-03
			RCC versus SKOV3	0.95	1.81E-05	5.70E-03
		10	A549 versus SKOV3	0.95	2.93E-05	2.48E-03
			Caco2 versus SKOV3	0.93	7.94E-05	2.12E-03
			H4 versus RCC	0.94	4.51E-05	5.22E-03
			H4 versus SKOV3	0.99	1.38E-07	1.79E-04
			RCC versus SKOV3	0.98	3.21E-07	2.74E-04
Serine	3.85 (dd); 3.95 (dd); 3.99 (dd)	10	H4 versus RCC	−0.91	2.32E-04	9.89E-03
			H4 versus SKOV3	−0.93	9.85E-05	2.41E-03
Succinate	2.41 (s)	5	H4 versus SKOV3	0.88	7.20E-04	4.12E-02
		10	Caco2 versus H4	−0.88	7.42E-04	1.87E-02
			H4 versus RCC	0.87	1.09E-03	1.97E-02
			H4 versus SKOV3	0.88	7.82E-04	9.72E-03
Taurine	3.25 (t); 3.41 (t)	5	Caco2 versus RCC	0.88	7.30E-04	2.48E-02
			H4 versus RCC	0.94	4.07E-05	2.62E-02
			RCC versus SKOV3	−0.96	1.02E-05	4.52E-03
		10	Caco2 versus RCC	0.95	1.81E-05	9.53E-03
			H4 versus RCC	0.84	2.09E-03	2.69E-02
Threonine	1.33 (d); 3.59 (d); 4.26 (m)	10	Caco2 versus H4	0.87	1.23E-03	2.67E-02
			H4 versus RCC	−0.84	2.14E-03	2.72E-02
Uridine	3.81 (d); 3.92 (d); 4.11 (m); 4.23 (t); 4.36 (t); 5.90 (d); 5.92 (d); 7.90 (d)	5	Caco2 versus RCC	−0.90	4.41E-04	2.05E-02
			H4 versus SKOV3	0.90	4.24E-04	3.07E-02
			RCC versus SKOV3	0.93	7.52E-05	1.06E-02
		10	H4 versus SKOV3	0.93	9.36E-05	2.35E-03
			RCC versus SKOV3	0.92	1.51E-04	4.73E-03
Valine	0.99 (d); 1.04 (d); 2.27 (m); 3.61 (d)	10	Caco2 versus H4	0.88	7.01E-04	1.81E-02
			H4 versus RCC	−0.91	2.56E-04	1.04E-02
			H4 versus SKOV3	−0.97	4.13E-06	4.22E-04

For each model (e.g. A vs. B), “+” indicates a higher correlation in B cells, whereas “–” indicates a higher correlation in A cells. *r* represents the correlation coefficient values; *P* represents significance level based on a two-tailed heteroscedastic *t*-test; *q* is corrected *P* values using Benjamini–Hochberg correction.

**Abbreviations:** bs, broad singlet; d, doublets; dd, double of doublets; m, multiplets; *n*, cell numbers; s, singlet; t, triplets; q, quartets (∼10^6^).

Statistically significant models were observed in the vast majority of pair-wise comparisons, except for A549 versus Caco2 (10 million) and A549 versus H4 (5 million). The model of A549 versus Caco2 from 5 million cell extracts was statistically significant, which was contributed by higher concentrations of phosphocholine, and decreased concentrations of glycine in Caco2 cells. However, the model of A549 versus RCC from 10 million rather than 5 million cell extracts was significant, corresponding to higher concentrations of formate, phosphocholine, and choline in RCC. The biochemical composition of A549 cells was also significantly different from SKOV3 cells. In the 5 million cell extracts model, the concentrations of phosphocholine, myo-inositol, glutathione, and glutamate were higher in SKOV3 compared with A549 cells. Additional metabolic differences, including higher concentrations of formate, uridine, lactate, and creatine, and lower concentrations of choline, acetate, and isoleucine, were observed in SKOV3 with 10 million cells compared with A549. The model of Caco2 versus H4 from 10 million cell extracts was statistically significant, with higher concentrations of threonine, valine and glycine, and lower concentrations of succinate in H4. The concentrations of formate, lactate, glutamate, glycine, myo-inositol, taurine, and glycerol phosphocholine was higher in both 5 and 10 million cell extracts of Caco2 compared with RCC. However, lower levels of uridine and phosphocholine and a higher level of choline were only observed in 5 and 10 million cell models, respectively. Higher concentrations of glutathione, myo-inositol, creatine, and lactate, and lower concentrations of choline in SKOV3 cells were observed to distinguish from Caco2 cells (both 5 and 10 million cells). While 5 million RCC cells showed higher concentrations of lactate, taurine, and glutamate in contrast with H4 cells, additional metabolites such as glycerol phosphocholine, phosphocholine, choline, succinate, threonine, serine, valine, and isoleucine were found to be different in concentrations in 10 million cells. Higher concentrations of uridine, glutathione, phosphocholine, lactate, myo-inositol, creatine, and succinate were observed in SKOV3 cells compared with H4 and RCC, whereas choline was found to be higher in RCC in comparison with SKOV3 (Figure 2).

### Metabolic characterization of A549, Caco2, H4, RCC, and SKOV3 cell culture media

High-intensity peaks present in the media samples were assigned based on 2D NMR spectra. These include acetate, acetone, alanine, citrate, formate, glucose, glutamate, glutamine, glycine, histidine, isoleucine, isopropanol, lactate, leucine, lysine, phenylalanine, pyroglutamate, pyruvate, succinate, threonine, tryptophan, tyrosine, and valine (Supplementary Figure S4).

**Figure 4:**
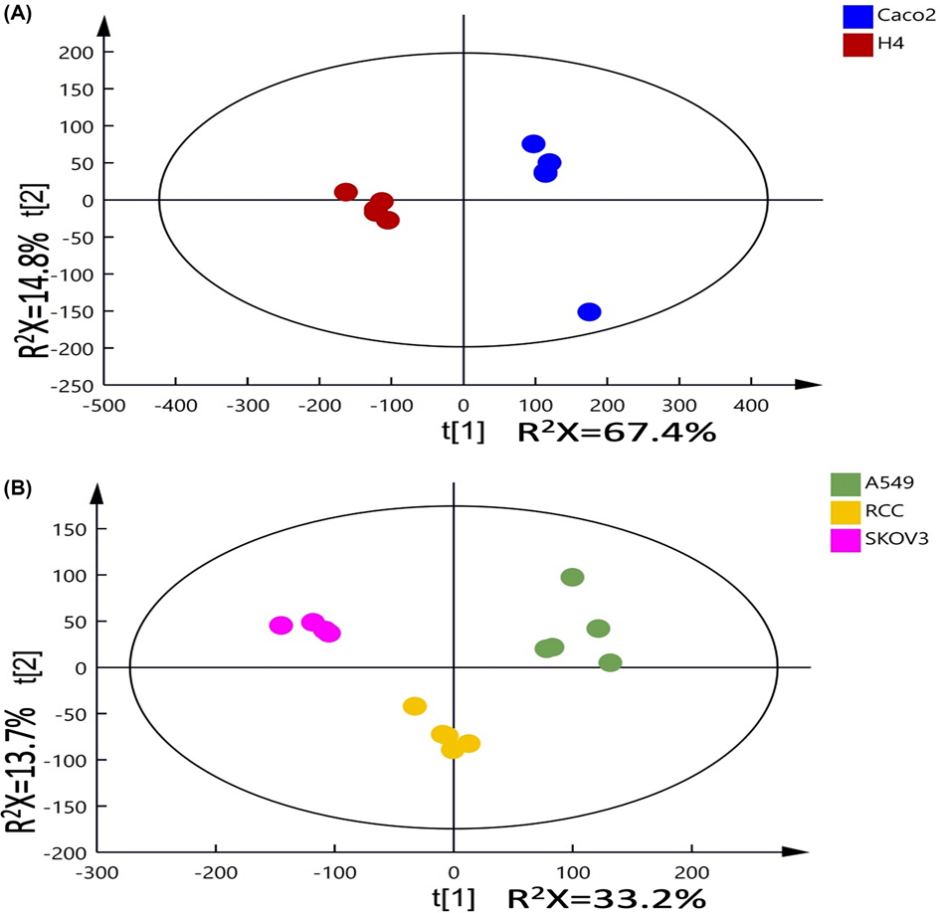
PCA scores plot of ^1^H-NMR spectra of media samples Principal components analysis (PCA) scores plot of ^1^H-NMR spectra of media samples ((**A**) DMEM culture media; (**B**) RPMI culture media) from 5 million cell culture with five replicates per cell type. R^2^X represents the fraction of variation in the NMR spectral data (R^2^X) modelled by each of the principal components.

^1^H-NMR spectral data from the cell culture media of these cancer cell lines were analyzed using PCA. As expected, the grouping pattern observed in the scores plots of media samples is based on the cell types (Supplementary Figure S5A), unlike the cell extracts where PC1 is dominated by the number of cells (Supplementary Figure S5B). Similar grouping patterns were observed from the PCA scores plots of all media samples, 5 or 10 million cell culture media samples. There is a clear separation along the PC1 between H4 and the other cell types, while a separation between Caco2 and the rest was observed along the PC2 (Supplementary Figure S5A,C and D). Given that both H4 and Caco2 were cultured using DMEM and the other three cell lines were cultured using RPMI, the major variation revealed by PCA was likely due to metabolic behavior of the H4 cells rather than the compositional differences between the two media. Additional PCA analyses were carried out for each type of media to compare the metabolic contribution of the cells to the media biochemical composition. The PCA scores plots based on the 5 million cell culture samples (Figure 4) show clear clustering based on the cell types. A similar pattern was also observed with 10 million cell culture media (Supplementary Figure S6).

**Figure 5:**
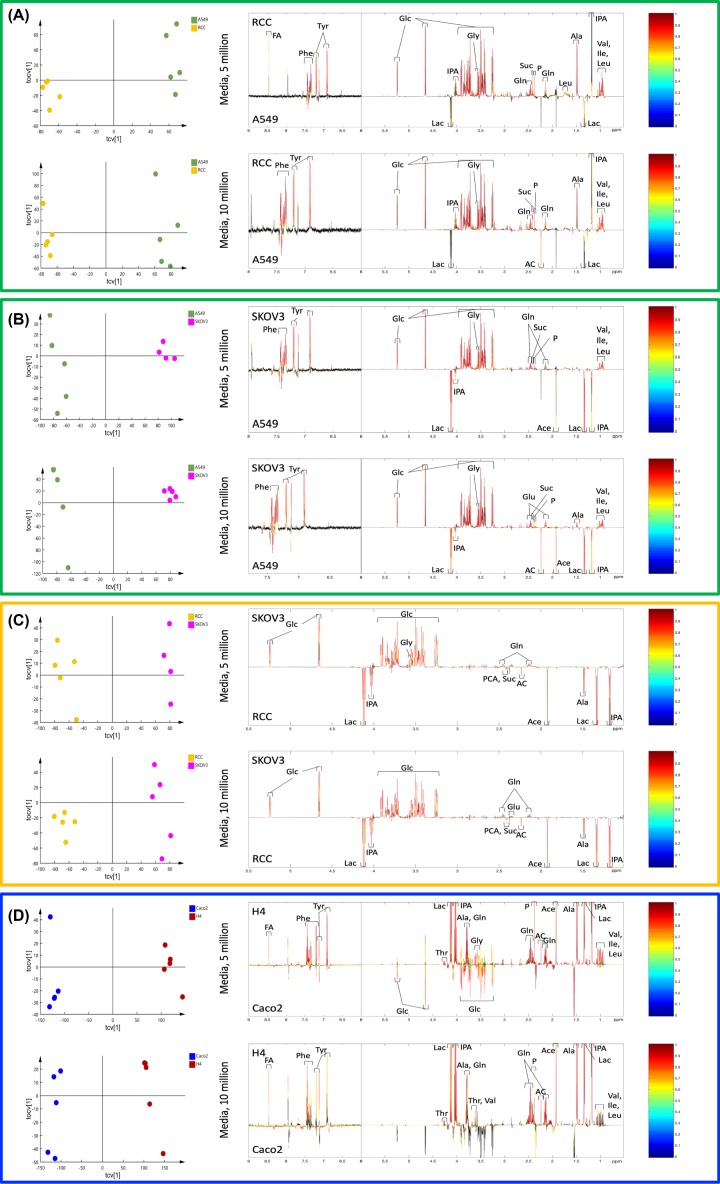
OPLS-DA cross-validated scores plots and loadings plots from ^1^H-NMR spectral data of the media samples Orthogonal projection to latent structures-discriminant analysis (OPLS-DA) loading plots from ^1^H-NMR spectral data of the media samples for comparison as A549 versus RCC (**A**), or SKOV3 (**B**); RCC versus SKOV3 (**C**); Caco2 versus H4 (**D**). Color bar indicates the correlation coefficient values (*r^2^*) to be high in red and low in blue. Unlike black peaks, colorful peaks are significant after Benjamini–Hochberg corrections.

**Figure 6:**
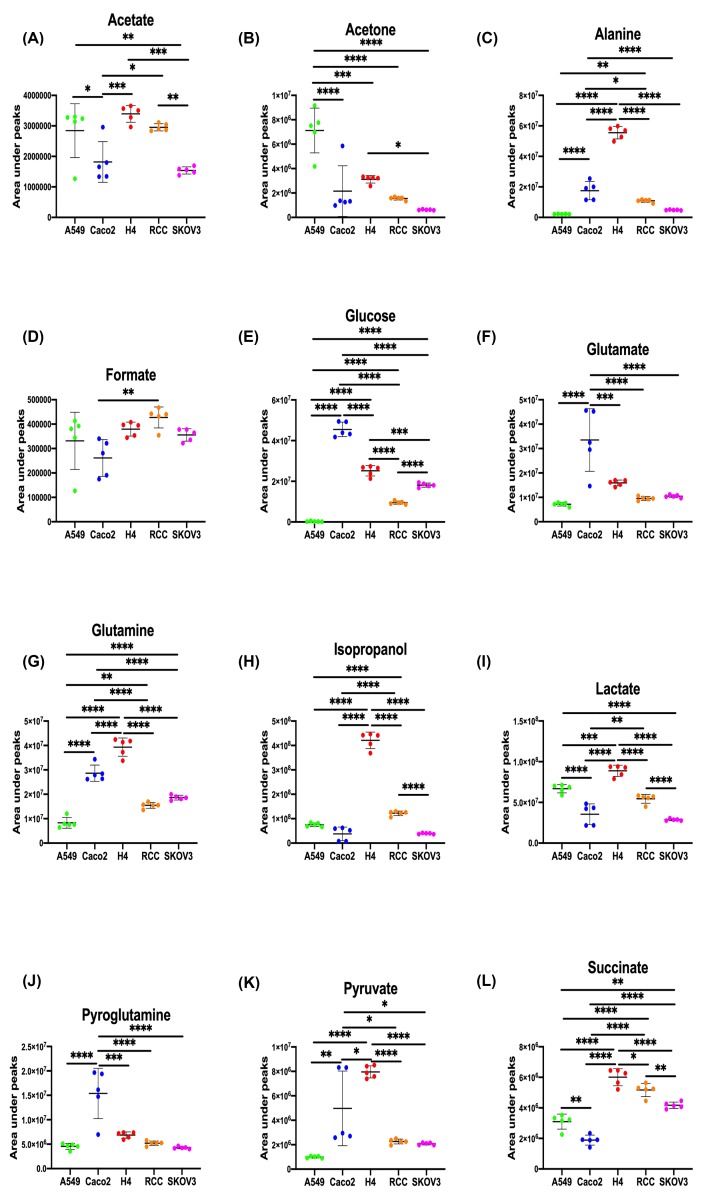
Peak integrals of the selected peaks from 12 metabolites from media samples of 10 million cells Peak integrals of the selected peaks from 12 metabolites from 10 million cells of A549, Caco2, H4, RCC, and SKOV3 media samples. The integrated values indicate relative concentrations of metabolites. (**A**) Acetate; (**B**) Acetone; (**C**) Alanine; (**D**) Formate; (**E**) Glucose; (**F**) Glutamate; (**G**) Glutamine; (**H**) Isopropanol; (**I**) Lactate; (**J**) Pyroglutamine; (**K**) Pyruvate; (**L**) Succinate. Data were shown as mean ± SD, with Tukey’s multiple comparisons test; *P<0.05, **P<0.01, ***P<0.001, ****P<0.0001 (*n*=5).

OPLS-DA models of media spectral data were calculated between different types of cells cultured in the same media and significant models were obtained from all comparisons (Table 3). OPLS-DA loadings plots and the metabolite changes observed in all pair-wise comparisons are shown in Figure 5 and Table 4. The peak integrals of 12 metabolites, identified from spectra of media samples cultured for 10 million cells of A549, Caco2, H4, RCC, and SKOV3, are presented in Figure 6.

**Table 3:** Summary of the parameters of the OPLS-DA models based on the media spectral data comparing different cell types cultured in the same media

Models	Cell number (million)	O-PLS-DA statistical parameters			
		R^2^X	R^2^Y	Q^2^Y	Permutation *P* value
A549 versus RCC	5	48.60%	99.86%	0.95	0.001
	10	50.20%	99.91%	0.95	0.001
A549 versus SKOV3	5	55.25%	99.86%	0.96	0.001
	10	55.42%	99.93%	0.96	0.001
RCC versus SKOV3	5	52.07%	99.85%	0.94	0.001
	10	41.75%	99.96%	0.94	0.001
Caco2 versus H4	5	81.85%	99.60%	0.98	0.005
	10	71.60%	99.50%	0.97	0.006

SKOV3 cell media (5 million) (*n*=4); other cell media (*n*=5). Permutation *P* values were derived from 1000 permutes.

**Table 4:** Summary of the metabolites that are present in different concentrations in the media with different cell types

Metabolites	δ^1^Η	*n*	Models	*r*	*P*	*q*
Acetate	1.92 (s)	5	A549 versus SKOV3	−0.91	6.30E-04	3.67E-03
			Caco2 versus H4	0.99	1.08E-10	2.89E-08
			RCC versus SKOV3	−0.99	8.64E-07	7.91E-05
		10	A549 versus SKOV3	−0.99	1.56E-07	3.19E-06
			Caco2 versus H4	0.99	3.53E-09	5.58E-07
			RCC versus SKOV3	−0.99	7.20E-09	1.06E-06
Acetone	2.24 (s)	5	Caco2 versus H4	0.98	1.59E-06	1.58E-05
			RCC versus SKOV3	−0.97	9.73E-06	3.15E-04
		10	A549 versus RCC	−0.98	1.14E-06	2.18E-05
			A549 versus SKOV3	−0.97	2.15E-06	2.50E-05
			Caco2 versus H4	0.96	7.20E-06	8.61E-05
			RCC versus SKOV3	−0.97	3.02E-06	6.35E-05
Alanine	1.48 (d); 3.78 (q)	5	A549 versus RCC	0.99	1.40E-11	5.30E-08
			Caco2 versus H4	0.99	5.80E-09	2.68E-07
		10	A549 versus RCC	0.99	6.40E-09	5.22E-07
			A549 versus SKOV3	0.99	1.36E-09	9.17E-08
			Caco2 versus H4	0.99	2.96E-10	1.03E-07
			RCC versus SKOV3	−0.99	1.38E-07	6.77E-06
Formate	8.46 (s)	5	A549 versus RCC	0.90	3.59E-04	2.59E-03
			Caco2 versus H4	0.99	1.69E-08	5.47E-07
		10	Caco2 versus H4	0.99	2.13E-07	7.73E-06
Glucose	3.25 (t); 3.39-3.55 (m); 3.69-3.93 (m); 4.65 (d); 5.24 (d)	5	A549 versus RCC	0.99	1.59E-10	1.28E-07
			A549 versus SKOV3	0.99	1.35E-11	4.45E-08
			Caco2 versus H4	−0.95	3.60E-05	1.83E-04
			RCC versus SKOV3	0.99	6.54E-09	5.05E-06
		10	A549 versus RCC	0.99	8.56E-12	2.86E-08
			A549 versus SKOV3	0.99	2.27E-12	1.36E-09
			RCC versus SKOV3	0.99	5.94E-12	1.90E-08
Glutamate	2.07 (m); 2.12 (m); 2.36(m); 3.77 (m)	10	RCC versus SKOV3	0.99	8.82E-09	1.19E-06
Glutamine	2.14 (m); 2.44 (m); 3.77 (m)	5	A549 versus RCC	0.99	8.32E-11	1.03E-07
			A549 versus SKOV3	0.99	1.19E-10	1.17E-07
			Caco2 versus H4	0.99	4.31E-11	2.33E-08
			RCC versus SKOV3	0.99	2.62E-07	3.72E-05
		10	A549 versus RCC	0.99	1.02E-09	1.75E-07
			A549 versus SKOV3	0.99	4.48E-11	7.65E-09
			Caco2 versus H4	0.99	1.30E-07	5.56E-06
			RCC versus SKOV3	0.99	8.77E-10	2.93E-07
Glycine	3.56 (s)	5	A549 versus RCC	0.98	5.59E-07	1.35E-05
			A549 versus SKOV3	0.99	4.06E-09	1.04E-06
			Caco2 versus H4	0.97	2.66E-06	2.35E-05
			RCC versus SKOV3	−0.89	1.36E-03	9.27E-03
		10	A549 versus RCC	0.99	6.91E-10	1.33E-07
			A549 versus SKOV3	0.99	1.44E-12	1.17E-09
Isoleucine	0.94 (t); 1.01 (d); 1.27 (m); 1.48 (m); 3.67 (m)	5	A549 versus RCC	0.98	2.79E-07	7.95E-06
			A549 versus SKOV3	0.99	1.73E-08	2.78E-06
			Caco2 versus H4	0.98	2.91E-07	4.30E-06
		10	A549 versus RCC	0.97	2.36E-06	3.81E-05
			A549 versus SKOV3	0.99	1.11E-09	7.91E-08
			Caco2 versus H4	0.92	1.54E-04	8.40E-04
Isopropanol	1.18 (dd); 4.03 (m)	5	A549 versus RCC	0.90	3.69E-04	2.66E-03
			A549 versus SKOV3	−0.91	6.49E-04	3.77E-03
			Caco2 versus H4	0.99	6.36E-09	2.83E-07
			RCC versus SKOV3	−0.99	5.65E-08	1.47E-05
		10	A549 versus RCC	0.95	2.25E-05	2.34E-04
			A549 versus SKOV3	−0.96	8.47E-06	7.66E-05
			Caco2 versus H4	0.99	2.20E-10	8.69E-08
			RCC versus SKOV3	−0.99	4.06E-09	7.88E-07
Lactate	1.33 (d); 4.11 (q)	5	A549 versus RCC	−0.86	1.34E-03	8.00E-03
			A549 versus SKOV3	−0.98	6.86E-06	1.27E-04
			Caco2 versus H4	0.99	2.19E-11	2.12E-08
			RCC versus SKOV3	−0.99	2.79E-07	3.80E-05
		10	A549 versus RCC	−0.83	3.07E-03	1.51E-02
			A549 versus SKOV3	−0.95	2.18E-05	1.70E-04
			Caco2 versus H4	0.99	6.74E-08	3.59E-06
			RCC versus SKOV3	−0.99	3.62E-08	2.96E-06
Leucine	0.96 (d); 0.97 (d); 1.69 (m); 1.71 (m); 3.74 (t)	5	A549 versus RCC	0.98	7.90E-07	1.75E-05
			A549 versus SKOV3	0.98	5.12E-06	1.03E-04
			Caco2 versus H4	0.98	7.64E-07	8.97E-06
		10	A549 versus RCC	0.98	9.12E-07	1.83E-05
			A549 versus SKOV3	0.98	1.51E-06	1.88E-05
			Caco2 versus H4	0.92	1.77E-04	9.28E-04
Phenylalanine	3.13 (dd); 3.28 (dd); 3.98 (dd); 7.33 (m); 7.38 (m); 7.43 (m)	5	A549 versus RCC	0.99	1.64E-10	1.28E-07
			A549 versus SKOV3	0.99	1.15E-07	8.43E-06
			Caco2 versus H4	0.98	2.38E-07	3.70E-06
		10	A549 versus RCC	0.99	5.80E-09	4.82E-07
			A549 versus SKOV3	0.99	8.15E-09	3.38E-07
			Caco2 versus H4	0.98	6.62E-07	1.63E-05
Pyroglutamate	2.04 (m); 2.41 (m); 2.51 (m); 4.18 (m)	5	RCC versus SKOV3	−0.96	3.50E-05	6.85E-04
		10	RCC versus SKOV3	−0.96	1.23E-05	1.88E-04
Pyruvate	2.38 (s)	5	A549 versus RCC	0.97	2.30E-06	3.84E-05
			A549 versus SKOV3	0.89	1.33E-03	6.72E-03
			Caco2 versus H4	0.99	1.09E-08	4.05E-07
		10	A549 versus RCC	0.98	1.20E-06	2.26E-05
			A549 versus SKOV3	0.92	1.86E-04	1.05E-03
			Caco2 versus H4	0.85	1.90E-03	5.00E-03
Succinate	2.41 (s)	5	A549 versus RCC	0.99	2.16E-08	1.34E-06
			A549 versus SKOV3	0.98	2.98E-06	7.16E-05
			RCC versus SKOV3	−0.97	1.22E-05	3.61E-04
		10	A549 versus RCC	0.95	3.25E-05	3.18E-04
			A549 versus SKOV3	0.99	1.03E-08	4.05E-07
			RCC versus SKOV3	-0.98	1.48E-06	3.72E-05
Threonine	1.33 (d); 3.59 (d); 4.26 (m)	5	Caco2 versus H4	0.98	2.38E-07	3.70E-06
		10	A549 versus RCC	0.93	1.07E-04	8.73E-04
			Caco2 versus H4	0.99	2.16E-09	4.00E-07
Tyrosine	3.06 (dd); 3.15 (dd); 3.94 (dd); 6.90 (d); 7.20 (d)	5	A549 versus RCC	0.96	1.28E-05	1.53E-04
			A549 versus SKOV3	0.96	5.49E-05	5.59E-04
			Caco2 versus H4	0.99	1.64E-07	2.78E-06
		10	A549 versus RCC	0.95	2.53E-05	2.58E-04
			A549 versus SKOV3	0.96	1.03E-05	8.99E-05
			Caco2 versus H4	0.92	1.49E-04	8.17E-04
Valine	0.99 (d); 1.04 (d); 2.27 (m); 3.61 (d)	5	A549 versus RCC	0.97	2.55E-06	4.16E-05
			A549 versus SKOV3	0.98	7.65E-06	1.36E-04
			Caco2 versus H4	0.98	2.82E-07	4.20E-06
		10	A549 versus RCC	0.96	8.42E-06	1.04E-04
			A549 versus SKOV3	0.98	2.76E-07	4.95E-06
			Caco2 versus H4	0.90	3.58E-04	1.53E-03

For each model (e.g. A vs. B), “+” indicates a higher correlation in B cells, whereas “–” indicates a higher correlation in A cells. *r* represents the correlation coefficient values; *P* represents significance level based on a two-tailed heteroscedastic *t*-test; *q* is corrected *P* values using Benjamini–Hochberg correction.

**Abbreviations:** bs, broad singlet; d, doublets; dd, double of doublets; m, multiplets; *n*, cell numbers; s, singlet; t, triplets; q, quartets (∼10^6^).

Lower concentrations of amino acids (e.g. phenylalanine, tyrosine, glycine, glutamine, alanine, valine, isoleucine, and leucine), glucose, and tricarboxylic acid (TCA) cycle intermediates (e.g. succinate and pyruvate) were observed in A549 compared with RCC or SKOV3, together with higher concentrations of lactate. Additionally, a higher concentration of alanine and a lower concentration of acetone were also found in a 10 million cell culture media (RPMI) of SKOV3, in contrast with A549. Metabolites present in the SKOV3 media that distinguish it from RCC include higher concentrations of glucose, glutamine, and lower concentrations of lactate, isopropanol, pyro glucose, succinate, acetone, acetate, and alanine.

Lower concentration of glucose was only observed in 5 million H4 cultured in DMEM compared with Caco2, whereas the increased concentrations of several metabolites were found in both 5 and 10 million models; these included formate, phenylalanine, tyrosine, threonine, lactate, isopropanol, alanine, glutamine, glycine, pyruvate, acetone, acetate, valine, isoleucine, and leucine (Figure 5).

## Discussion

The present study was based on ^1^H-NMR spectroscopy-based metabolic phenotyping and demonstrated the significant metabolic differences among five cell lines, namely, lung (A549), colonic (Caco2), brain (H4), renal (RCC), and ovarian (SKOV3) cancer cells. The intra-group variation was higher in the H4 cell line in contrast with the others, particularly when cultured as 10 million cells. It is likely that H4 cell growth are more sensitive to environmental conditions, and the cell growth rate and metabolic behavior may likely be affected with subtle changes in culture condition. One of the most profound findings with regards to metabolic changes was the significantly higher intracellular concentrations of glutathione, myo-inositol, and phosphocholine in SKOV3, compared with other cell lines. Glutathione (GSH) is the most abundant non-protein thiol, which functions as an antioxidant and a redox regulator. It has been found that stem cells required high levels of GSH to maintain stem cell function and migration capabilities *in vitro* [9]. Similarly, GSH plays an important role in cancer progression and resistance to therapy. Indeed, it is reported to be associated with chemoresistance to platinum salts, which is one of the main treatments for ovarian cancer [10]. Myo-inositol and phosphocholine (ChoP) have been reported in SKOV3 cells and their cellular concentrations reduced after treatment of Ptac2S, a novel anticancer agent [11].

Glutamate was found to be higher in SKOV3 and RCC cells compared with other cell lines. Glutamate is an amino acid that plays a key role in energy and carbon metabolism and synthesis of amino acids and nucleotides for all cells. In cancer cells, glucose-based glycolysis and glutamate-based glutaminolysis are major two ways for ATP production. With the high levels of glutamate, glioma cells can be rescued from death [12].

Another key finding was the high abundance of amino acids in the H4 cells; amino acids presented in higher levels in H4 versus other cell lines include serine, methionine, threonine, valine, glycine, and acetate. The function of mitochondria is largely dependant on the pathway of serine to formate, which is then released into the cytoplasm to contribute to nucleotide synthesis [13]. As the level of serine was higher in the H4 cell line compared with other cell lines, the mitochondrial function of H4 cells may be disturbed. Amino acids and acetate in cancers can be used as nutritional supports for protein synthesis and lipid metabolism, respectively. Threonine was reported to be responsible for Akt and ERK signalling pathway in breast cancer [14].

It has been found in our study that the concentrations of choline, creatine, lactate, alanine, fumarate, and succinate varied significantly among different cell types. Ovarian cancer cell line exhibited the highest levels of alanine, lactate, and TCA cycle intermediates (e.g. succinate, fumarate) and methanol, and the lowest levels of choline, whereas lung cancer cell line showed the lowest levels of lactate and alanine. Lactate has been reported to be elevated in the cancerous cells due to lactic acidosis and glucose deprivation [15], while glutamine and alanine metabolism may be altered in breast cancer [16]. Due to ‘Warburg effects’, in cancer cells (especially cancerous tumors), the oxidative phosphorylation pathway is more likely to shift to glycolysis, which increases the level of lactate and decreases the level of TCA cycle intermediates [17]. Alanine can be a fuel source for the TCA cycle; increased alanine levels could indicate decreased activity of the TCA cycle, which could be attributed to the higher Warburg effects observed in SKOV3 cells [18]. Choline may affect the progression of cancer through one-carbon metabolism. Therefore, higher malignancy of SKOV3 cells could be due to an accelerated one-carbon metabolism and thus decreased levels of choline [19].

Media concentrations of isopropanol was found to be the highest in RCC, followed by A549 and SKOV3, while acetone was the highest in A549, followed by RCC and SKOV3, together with a higher level in H4 compared with Caco2. Interestingly, isopropanol and acetone were deemed as a potential biomarker in a series of diseases including cancer [20,21]. There is a reversible reaction between acetone and isopropanol under the action of alcohol dehydrogenase [22]. It was reported that under the circumstances of starvation and a ketogenic diet, ketone bodies such as acetone is produced [23]. Additionally, higher concentrations of breath acetone were also reported in lung cancer patients, which is in line with our previous findings of the highest acetone production in lung cancer cells [21].

The concentrations of alanine in the culture media were the highest in the RCC, followed by SKOV3 and A549. A previous study showed that renal cell carcinoma results in an increased level of alanine in cells, which might be due to the downregulated expression of *ALDH6A1* gene [24], thus demonstrating that the *ALDH6A1* gene may encode methylmalonate semialdehyde dehydrogenase, was deficient and hence the level of alanine was increased.

## Conclusion

Our study showed that ^1^H-NMR-based metabolic phenotyping analysis can detect the cellular metabolic profile in five different cancer cell types, including lung, colonic, brain, renal, and ovarian cancers. Similarly, their metabolic profiles can also be measured in culture media. It may be concluded that ^1^H-NMR-based metabolic phenotyping can be used to detect cellular metabolisms of different cancerous cells and improve our understanding of the metabolism of certain cancers.

## Supplementary Material

Supplementary Figures S1-S6 and Table S1

## Abbreviations

2-D: two-dimensional
ChoP: phosphocholine
COSY: correlation spectroscopy
D_2_O: deuterium oxide
GSH: Glutathione
HSQC: heteronuclear single quantum coherence spectroscopy
NaN_3_: sodium azide
NMR: nuclear magnetic resonance
PC: principal components
TCA: tricarboxylic acid
TOCSY: total correlation spectroscopy
TSP: 3-(trimethylsilyl)-[2,2,3,3-^2^H_4_]-propionic acid sodium salt
