# Supplementary material for: A biochemical comparison of the lung, colonic, brain, renal, and ovarian cancer cell lines using 1H-NMR spectroscopy
Source: Biosci Rep. 2020 Apr 23;40(4):BSR20194027. doi: 10.1042/BSR20194027 (PMC7198042; doi:10.1042/BSR20194027)
Supplement: Supplementary Figures S1-S6 and Table S1 [file BSR-2019-4027_supp.pdf]

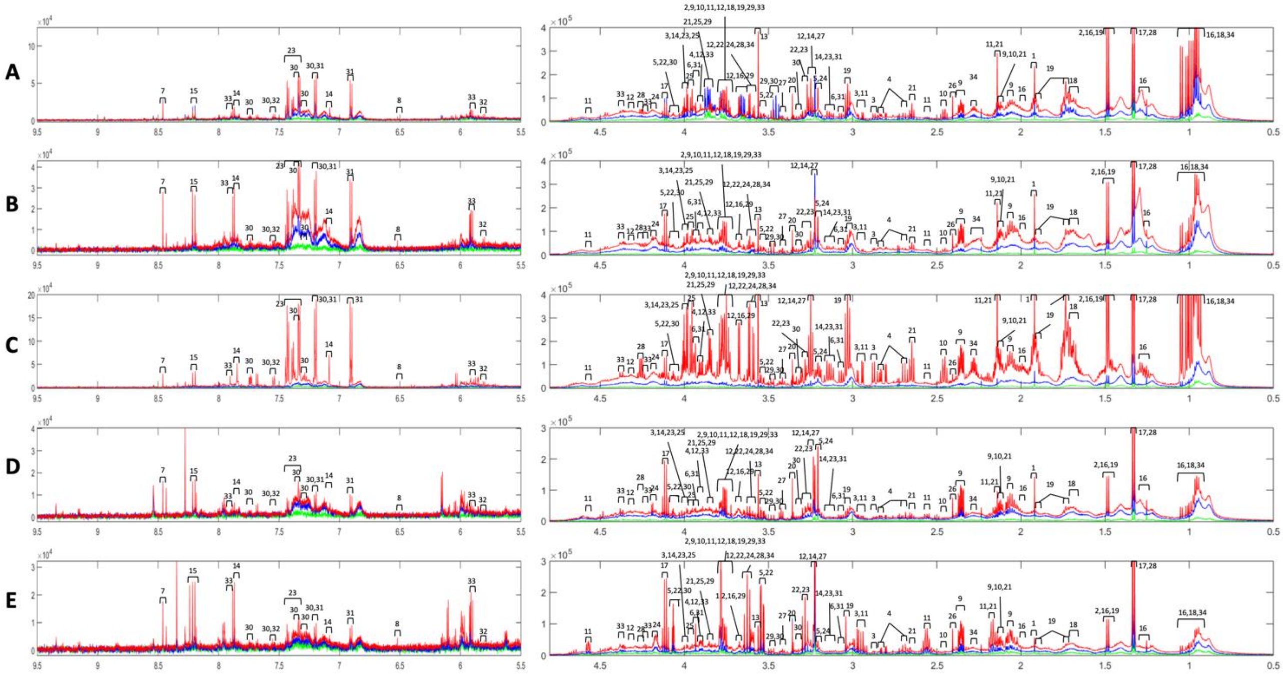
A
B
C
D
E

Figure S1. Median ^{1}H-NMR spectra of A549 (A), Caco2 (B), H4 (C), RCC (D), and SKOV3 (E) cell extracts from 1 (green), 5 (blue) and 10 (red) million cells. Keys: 1, Acetate; 2, Alanine; 3, Asparagine; 4, Aspartate; 5, Choline; 6, Creatine; 7, Formate; 8, Fumarate; 9, Glutamate; 10, Glutamine; 11, Glutathione; 12, Glycerol phosphocholine; 13, Glycine; 14, Histidine; 15, Hypoxanthine; 16, Isoleucine; 17, Lactate; 18, Leucine; 19, Lysine; 20, Methanol; 21, Methionine; 22, Myo-inositol; 23, Phenylalanine; 24, Phosphocholine; 25, Serine; 26, Succinate; 27, Taurine; 28, Threonine; 29, Trehalose; 30, Tryptophan; 31, Tyrosine; 32, Uracil; 33, Uridine; 34, Valine.

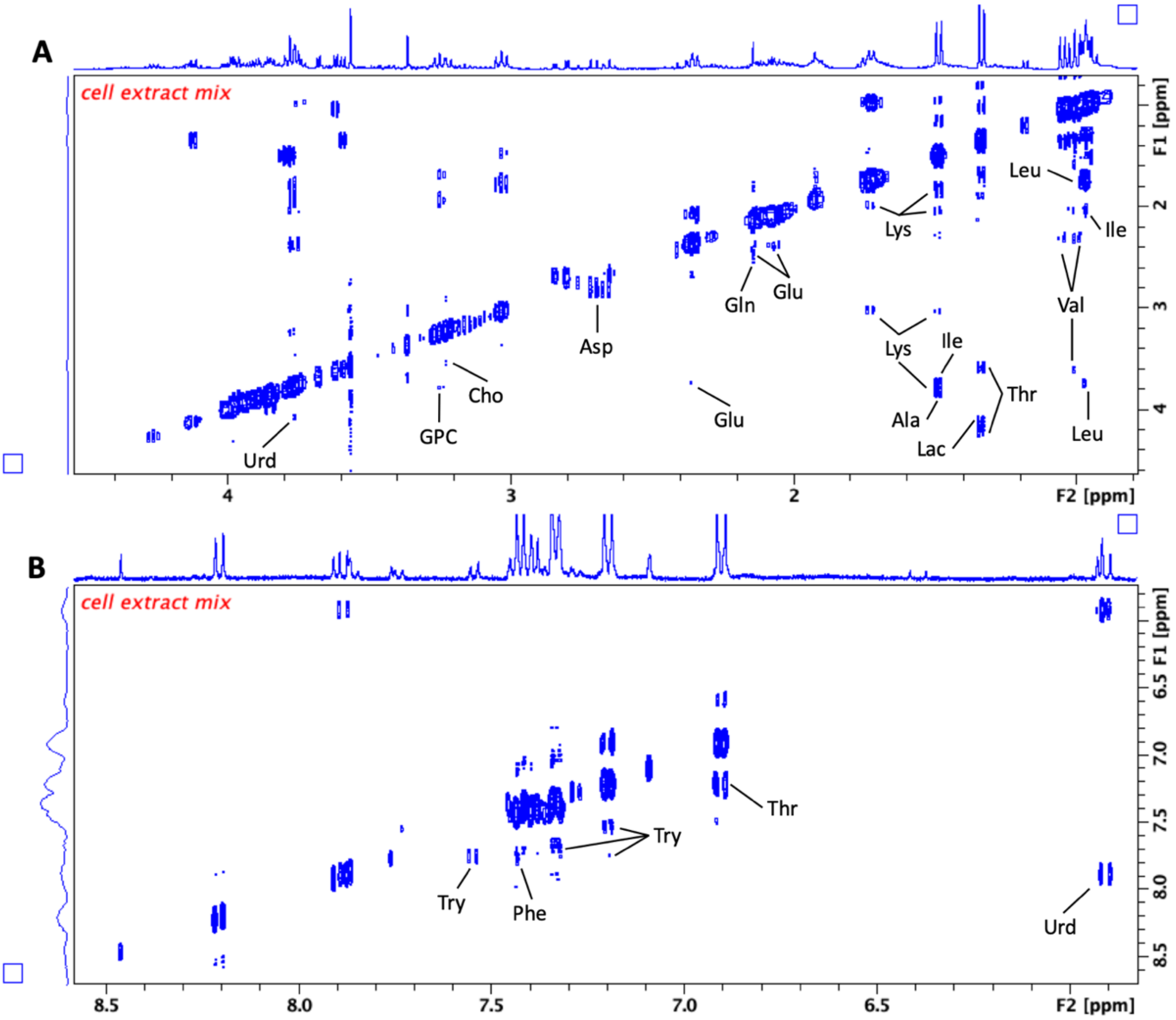
A
cell extract mix
Leu
Ile
Lys
Val
Gln
Glu
Asp
Cho
GPC
Urd
Ile
Lys
Thr
Ala
Lac
Leu
F1 [ppm]
F2 [ppm]
B
cell extract mix
Thr
Try
Try
Phe
Urd
F1 [ppm]
F2 [ppm]

Figure S2. Typical ^{1}H-^{1}H total correlation spectroscopy (TOCSY) two-dimensional (2D) NMR data from cell extracts for metabolite identification.

A. $\delta^{1}H$ (ppm) range between 0.8-4.6; B. $\delta^{1}H$ (ppm) range between 5.8-8.6. Ala, alanine; Asp, aspartate; Cho, choline; Glu, glutamate; Gln, glutamine; GPC, glycerol phosphocholine; Ile, isoleucine; Lac, lactate; Leu, leucine; Lys, lysine; Phe, phenylalanine; Thr, threonine; Try, tryptophan; Urd, uridine; Val, valine.

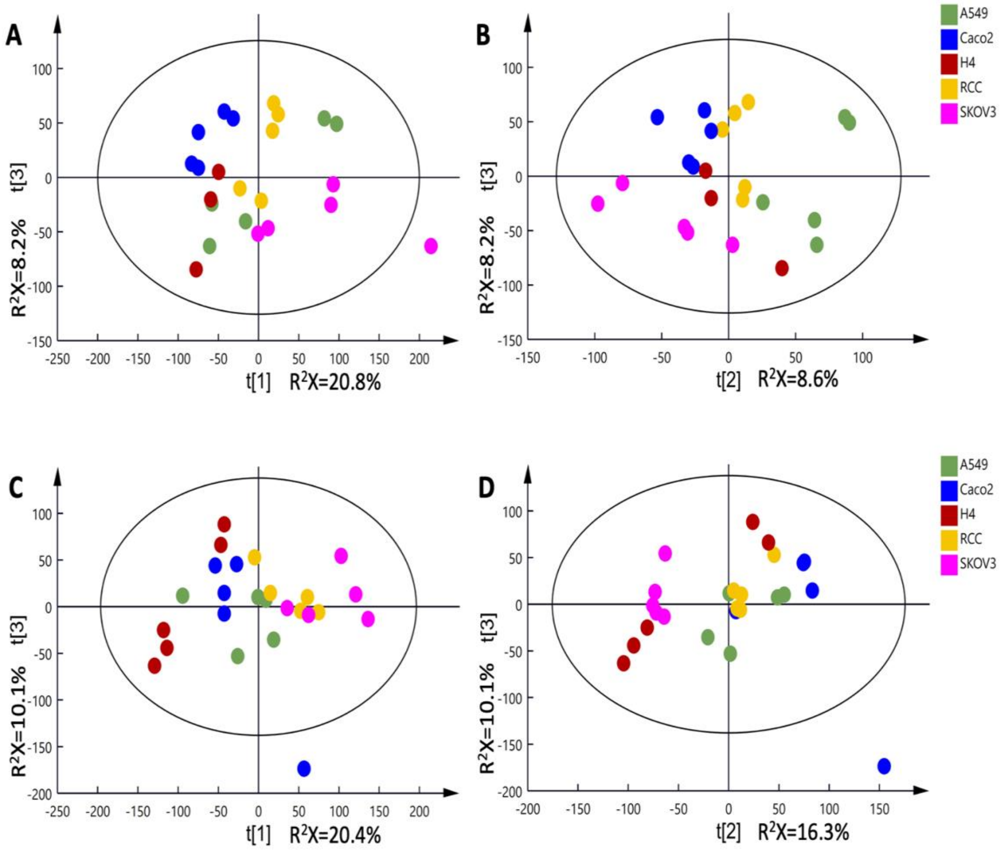
A
B
C
D
A549
Caco2
H4
RCC
SKOV3
t[1] R^2X=20.8%
t[3] R^2X=8.2%
t[2] R^2X=8.6%
t[3] R^2X=8.2%
t[1] R^2X=20.4%
t[3] R^2X=10.1%
t[2] R^2X=16.3%
t[3] R^2X=10.1%

Figure S3. Principal components analysis (PCA) scores plots of ^{1}H-NMR spectra of cellular extracts from 5 (A, t[1] vs. t[3]; B, t[2] vs. t[3]) and 10 (C, t[1] vs. t[3]; D, t[2] vs. t[3]) million cells with 5 replicates per cell type. R^2X represents the fraction of variation in the NMR spectral data (R^2X) modelled by each of the principal components. t[1], t[2] and t[3] are the 1st, 2nd and 3rd principal component.

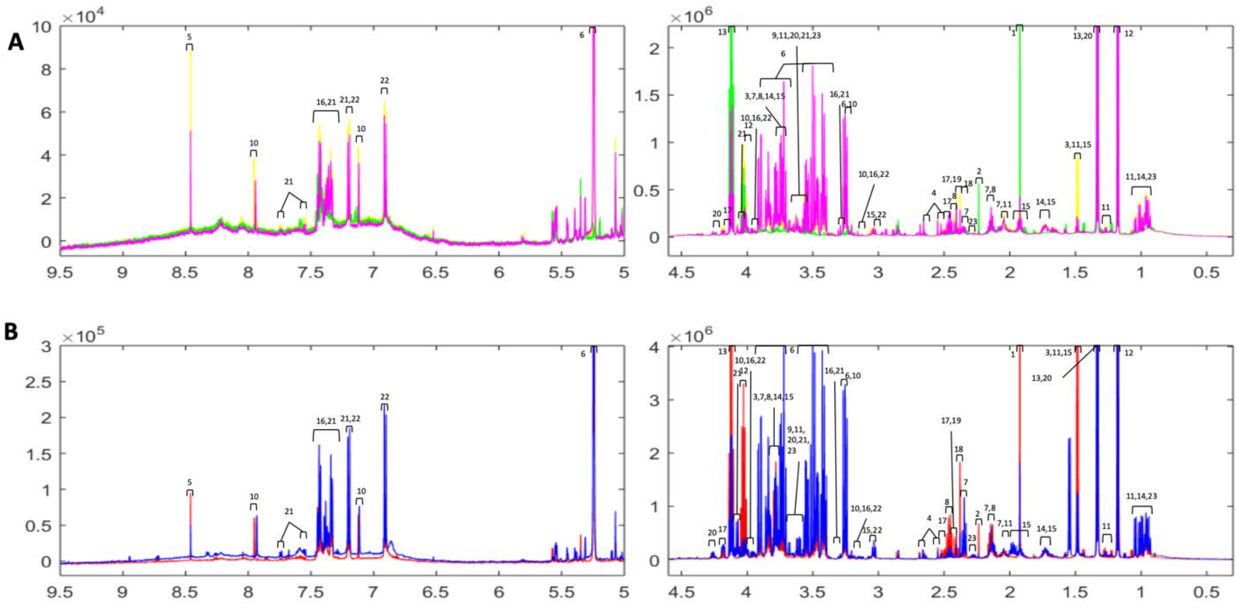
A
B
×10^4
×10^6
×10^5
9.5 9 8.5 8 7.5 7 6.5 6 5.5 5
4.5 4 3.5 3 2.5 2 1.5 1 0.5
16,21
21,22
22
10
5
6
13
1
13,20
12
3,11,15
11,14,23
9,11,20,21,23
3,7,8,14,15
10,16,22
16,21
6,10
15,22
17,19
14,15
7,11
7,8
18

Figure S4. 600 MHz ^{1}H-NMR spectra of RPMI (A) and DMEM (B) culture media obtain from A549 (green), RCC (yellow), SKOV3 (pink), H4 (red) and Caco2 (blue) cell culture experiments (5 million cells were present). Keys: 1, Acetate; 2, Acetone; 3, Alanine; 4, Citrate; 5, Formate; 6, Glucose; 7, Glutamate; 8, Glutamine; 9, Glycine; 10, Histidine; 11, Isoleucine; 12, Isopropanol; 13, Lactate; 14, Leucine; 15, Lysine; 16, Phenylalanine; 17, Pyroglutamate; 18, Pyruvate; 19, Succinate; 20, Threonine; 21, Tryptophan; 22, Tyrosine; 23, Valine.

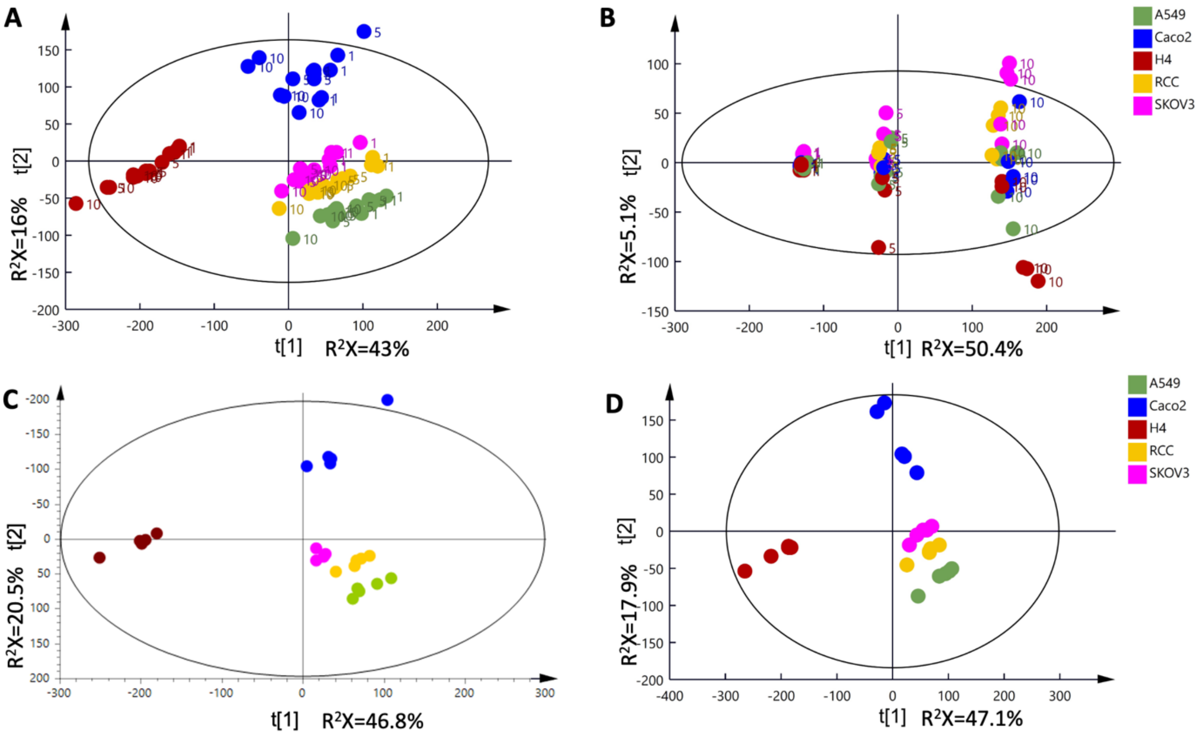
A
B
C
D
A549
Caco2
H4
RCC
SKOV3
t[1] R²X=43%
t[2] R²X=16%
t[1] R²X=50.4%
t[2] R²X=5.1%
t[1] R²X=46.8%
t[2] R²X=20.5%
t[1] R²X=47.1%
t[2] R²X=17.9%

Figure S5. Principal components analysis (PCA) scores plots of ^{1}H-NMR spectra of media samples (A) and cell extracts (B) from 1, 5 and 10 million cell culture with 5 replicates per cell type. PCA scores plots of ^{1}H-NMR spectra of media samples from 5 (C) and 10 (D) million cell culture with 5 replicates per cell type. R^2X represents the fraction of variation in the NMR spectral data (R^2X) modelled by each of the principal components. The labels of the dots represent the cell number.

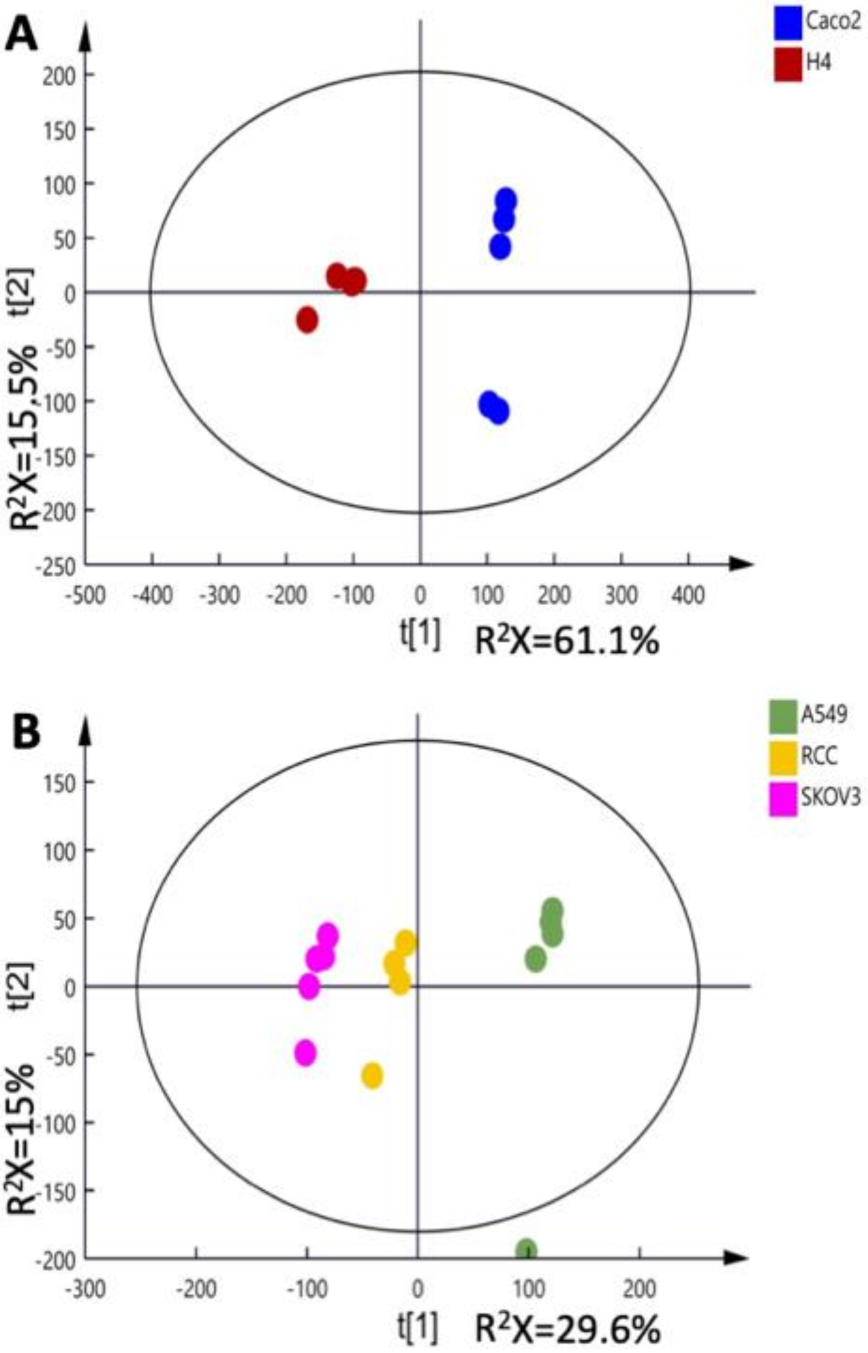
A
Caco2
H4
R²X=15.5% t[2]
t[1] R²X=61.1%
B
A549
RCC
SKOV3
R²X=15% t[2]
t[1] R²X=29.6%

Figure S6. Principal components analysis (PCA) scores plot of ^{1}H-NMR spectra of media samples (A, DMEM culture media; B, RPMI culture media) from 10 million cell culture with 5 replicates per cell type. R^2X represents the fraction of variation in the NMR spectral data (R^2X) modelled by each of the principal components.

Table S1. Metabolites assignment from cell (C) and media (M) sample.

Metabolites	δ^1H (multiplicities)	Sample type
Acetate (Ace)	1.92 (s)	C, M
Acetone (AC)	2.24 (s)	M
Alanine (Ala)	1.48 (d); 3.78 (q)	C, M
Asparagine (Asn)	2.86 (dd); 2.96 (dd); 4.00 (dd)	C
Aspartate (Asp)	2.69 (m); 2.82 (dd); 3.90 (m)	C
Choline (Cho)	3.21 (s); 3.53 (m); 4.07 (m)	C
Citrate (Cit)	2.53 (dd); 2.69 (dd)	M
Creatine (Cre)	3.04 (s); 3.93 (s)	C
Formate (FA)	8.46 (s)	C, M
Fumarate (Fum)	6.52 (s)	C
Glucose (Glc)	3.25 (t); 3.39-3.55 (m); 3.69-3.93 (m); 4.65 (d); 5.24 (d)	M
Glutamate (Glu)	2.07 (m); 2.12 (m); 2.36 (m); 3.77 (m)	C, M
Glutamine (Gln)	2.14 (m); 2.44 (m); 3.77 (m)	C, M
Glutathione (GSH)	2.17 (m); 2.56 (m); 2.96 (m); 3.78 (m); 4.56 (t)	C
Glycerol phosphocholine (GPC)	3.23 (s); 3.60 (dd); 3.68 (t); 3.72 (dd); 3.89 (m); 4.32 (t)	C
Glycine (Gly)	3.56 (s)	C, M
Histidine (His)	3.14 (dd); 3.25 (dd); 3.99 (dd); 7.08 (s); 7.84 (s)	C, M
Hypoxanthine (Hyp)	8.20 (s); 8.21 (s)	C
Isoleucine (Ile)	0.94 (t); 1.01 (d); 1.27 (m); 1.48 (m); 1.99 (m); 3.67 (m)	C, M
Isopropanol (IPA)	1.18 (dd); 4.03 (m)	M
Lactate (Lac)	1.33 (d); 4.11 (q)	C, M
Leucine (Leu)	0.96 (d); 0.97 (d); 1.69 (m); 1.71 (m); 3.74 (t)	C, M
Lysine (Lys)	1.48 (m); 1.72 (m); 1.91 (m); 3.03 (t); 3.76 (t)	C, M
Methanol (MeOH)	3.36 (s)	C
Methionine (Met)	2.14 (s); 2.16 (m); 2.65 (t); 3.86 (m)	C
Myo-inositol (MI)	3.28 (t); 3.53 (dd); 3.62 (t); 4.06 (t)	C
Phenylalanine (Phe)	3.13 (dd); 3.28 (dd); 3.98 (dd); 7.33 (m); 7.38 (m); 7.43 (m)	C, M
Phosphocholine (ChoP)	3.22 (s); 3.60 (m); 4.17 (m)	C
Pyroglutamate (PCA)	2.04 (m); 2.41 (m); 2.51 (m); 4.18 (m)	M
Pyruvate (P)	2.38 (s)	M
Serine (Ser)	3.85 (dd); 3.95 (dd); 3.99 (dd)	C
Succinate (Suc)	2.41 (s)	C, M
Taurine (Taur)	3.25 (t); 3.41 (t)	C
Threonine (Thr)	1.33 (d); 3.59 (d); 4.26 (m)	C, M
Trehalose (Tre)	3.46 (m); 3.67 (m); 3.76 (m); 3.85 (m); 5.20 (d)	C
Tryptophan (Trp)	3.32 (m); 3.49 (m); 4.05 (m); 7.20 (t); 7.29 (t); 7.33 (s); 7.54 (d); 7.74 (d)	C, M
Tyrosine (Tyr)	3.06 (dd); 3.15 (dd); 3.94 (dd); 6.90 (d); 7.20 (d)	C, M
Uracil (Ura)	5.81 (d); 7.54 (d)	C
Uridine (Urd)	3.81 (d); 3.92 (d); 4.11 (m); 4.23 (t); 4.36 (t); 5.90 (d); 5.92 (d); 7.90 (d)	C
Valine (Val)	0.99 (d); 1.04 (d); 2.27 (m); 3.61 (d)	C, M

Abbreviation: S, singlet; d, doublets; t, triplets; q, quartets; dd, double of doublets; m, multiplets.
